# Cancer based pharmacogenomics network supported with scientific evidences: from the view of drug repurposing

**DOI:** 10.1186/s13040-015-0042-8

**Published:** 2015-02-25

**Authors:** Liwei Wang, Hongfang Liu, Christopher G Chute, Qian Zhu

**Affiliations:** 1 Department of Medical Informatics, School of Public Health, Jilin University, Changchun, 130021 China; 2 Department of Health Science Research, Mayo Clinic, Rochester, MN 55901 USA; 3 Division of General Internal Medicine, Johns Hopkins University, Baltimore, MD 21287 USA; 4 Department of Information Systems, University of Maryland Baltimore County, Baltimore, MD 21055 USA

**Keywords:** Pharmacogenomics, Cancer, Network, Drug repurposing

## Abstract

**Background:**

Pharmacogenomics (PGx) as an emerging field, is poised to change the way we practice medicine and deliver health care by customizing drug therapies on the basis of each patient’s genetic makeup. A large volume of PGx data including information among drugs, genes, and single nucleotide polymorphisms (SNPs) has been accumulated. Normalized and integrated PGx information could facilitate revelation of hidden relationships among drug treatments, genomic variations, and phenotype traits to better support drug discovery and next generation of treatment.

**Methods:**

In this study, we generated a normalized and scientific evidence supported cancer based PGx network (CPN) by integrating cancer related PGx information from multiple well-known PGx resources including the Pharmacogenomics Knowledge Base (PharmGKB), the FDA PGx Biomarkers in Drug Labeling, and the Catalog of Published Genome-Wide Association Studies (GWAS). We successfully demonstrated the capability of the CPN for drug repurposing by conducting two case studies.

**Conclusions:**

The CPN established in this study offers comprehensive cancer based PGx information to support cancer orientated research, especially for drug repurposing.

## Background

In 2003, the US Food and Drug Administration (FDA) recognized the importance of PGx data for the evaluation of drug safety and efficacy by starting a voluntary data exchange program, which requests that pharmaceutical companies submit genomic data along with their new drug packages. So far, the FDA has documented PGx information for more than 100 drugs associated with more than 50 genes [1]. Of these drugs, 42 FDA cancer drugs include PGx information in their package inserts. Clearly, cancer therapy is one of the most intensively studied topics in PGx [2-4], and relevant PGx data are accumulating quickly. Thus, it is critical to determine how to use and integrate cancer based PGx information effectively, thereby revealing hidden relationships among drug treatments, genomic variations, and phenotype traits and better supporting drug discovery and next generation of treatment. To our knowledge, no integration efforts have been directed specifically toward cancer based PGx. Suggested Ontology for Pharmacogenomics (SO-Pharm) [5] and Pharmacogenomics Ontology (PO) [6] are two existing ontologies for general PGx integration. They provided a first step toward integrating and representing PGx (and related) knowledge in the web ontology language (OWL), a web standard [7]. SO-Pharm contains so many classes and relations to represent generic PGx information that it is computationally expensive “and leads to significantly higher complexity for knowledge composition” [5]. It therefore presents challenges to users “in asserting knowledge or making routine queries” [5]. PO is a case-driven PGx data integration platform that aims to question-answering. Our study aims to integrate PGx information by focusing on oncology domain from diverse PGx resources. In addition, we will not only integrate existing PGx information, but also add inferred associations, which will support the novel indication detection for used drugs.

Idiosyncratic information without semantic interoperability and standard-based annotation, however, adds no value to the scientific commons. These idiosyncratic data must be annotated using standard terms and elements that correspond to the way scientists might search, integrate, inference, or expand upon the data. In the oncology community, the FDA and National Cancer Institute (NCI) attempt to document approved cancer drug information in a meaningful way. For instance, cancer drugs can be browsed by approved date with detailed description from the FDA [8]; they also can be queried/browsed by specific cancer type from the NCI [9], in which cancer drugs have been mapped to the NCI Thesaurus [2]. Nevertheless, to our knowledge, there is no data normalization effort made for cancer based PGx information. Lack of such effort hinders data sharing and further data integration. The CPN constructed in this study has been highlighted with normalization tags by leveraging the controlled terminologies and vocabularies.

In this study, we integrated multiple well known PGx resources including the PharmGKB [1], the FDA Pharmacogenomic Biomarkers in Drug Labeling [10] and the Catalog of Published Genome-Wide Association Studies [11], and represented terms by using relevant standards to construct a cancer based PGx network, named CPN (Cancer based PGx Network). This work was intended to demonstrate the feasibility of constructing the CPN to support possible drug repurposing candidate identification. To illustrate the capability of the CPN for drug repurposing, two case studies have been performed successfully.

## Materials

### NCI cancer list

National Cancer Institute (NCI) has maintained the alphabet links for information on a particular type of cancer. In this study, we have manually collected 160 distinct cancer types through de-duplication including bladder cancer, breast cancer, leukemia, and so on from NCI by Nov 14, 2013 [12].

### Pharmacogenomics knowledge base (PharmGKB)

PharmGKB contains genomic, phenotype and clinical information collected from PGx studies. It provides information regarding variant annotations, drug-centered pathway, pharmacogenomic summaries, clinical annotations, PGx-based drug-dosing guidelines, and drug labels with PGx information [1]. In this study, we used PGx information extracted from a relationship file received from the PharmGKB by May 8, 2013, which provides associations between two PGx concepts, including drug, gene, disease, SNP and haplotype. Some examples are shown in Table 1. All fields listed in Table 1 were extracted and applied in this study.

**Table 1:** **Examples of PGx associations extracted from the PharmGKB**

Entity1_id	Entity1_ name	Entity1_ type	Entity2_id	Entity2_ name	Entity2_ type	PMIDs
PA443512	Urinary bladder neoplasms	Disease	rs762551	rs762551	Variant location	18798002
rs762551	rs762551	Variant location	PA443434	Arthritis, Rheumatoid	Disease	18496682
PA443434	Arthritis, Rheumatoid	Disease	PA27093	CYP1A2	Gene	18496682;19581389
PA27093	CYP1A2	Gene	PA450688	olanzapine	Drug	19636338;21519338

The detailed information about individual disease, drug and gene terms were extracted from the corresponding *Disease*, *Drug* and *Gene* files downloaded from the PharmGKB by November 15, 2013 [13].

### FDA Pharmacogenomic biomarkers in drug labeling

The US Food and Drug Administration (FDA) provides a table of biomarkers for some FDA-approved drugs. The table contains “Therapeutic areas” field indicating the treatment intention of the drugs, such as “Oncology”, “Psychiatry”, etc., as well as the “HUGO Symbol” field representing associated genes. In this study we extracted these two fields that are “Oncology” related. The table was downloaded by Dec 3, 2013 [8].

### Catalog of published genome-wide association studies

NIH provides a Catalog of Published Genome-Wide Association Studies (GWAS), which has identified single nucleotide polymorphisms (SNPs) and reported genes for major disease traits. We extracted cancers and related genes and SNPs from the “Disease/Trait”, the “Reported Gene(s)” and “SNPs” fields respectively. The Catalog was downloaded by Dec 3, 2013 [11].

### National Center for Biomedical Ontology (NCBO)

The NCBO provides an ontology-based web service that can annotate public datasets with biomedical ontology concepts [14]. The reasons to select the NCBO bioportal for the normalization task in this study are 1) our previous work [15] has shown the capability of NCBO to support PGx data normalization, 2) its convenience of online access and its large scale of more than 400 ontologies [16] beyond other tools such as Metamap. We used the NCBO Bioportal REST service [17] to access biomedical ontologies. In this study, we utilized this service to normalize disease and drug terms with Systematized Nomenclature of Medicine-Clinical Terms (SNOMED-CT) [18] and RxNorm [19].

### SemMedDB

SemMedDB is a repository of semantic predications (subject-predicate-object triples) extracted from the entire set of PubMed citations with SemRep. The subject and object pair corresponds to UMLS Metathesaurus concepts, and the predicate to a relation type in an extended version of the semantic network. SemMedDB contains eight tables and is updated at regular intervals. We downloaded the latest PREDICATION_AGGREGATE table with ending date of MAR 31 2014 [20]. In this study, we identified scientific evidence, PubMed IDs from SemMedDB for PGx associations present in the CPN.

## Methods

**Figure 1:**
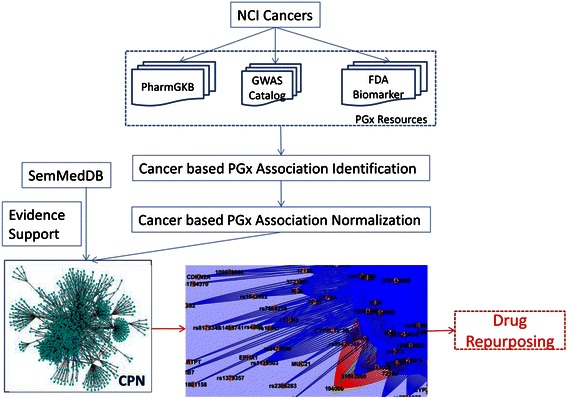
**The architecture of the approach being used for the CPN construction.**

In this study, we designed an approach including four steps to generate the CPN: 1) cancer based PGx association identification, 2) cancer based PGx concept normalization, 3) scientific evidence identification, and 4) the CPN generation. In the first step, we identified cancer based PGx associations from the PharmGKB, the GWAS Catalog and the FDA Biomarker table. Then we mapped cancer based PGx concepts to standard vocabularies, for instance, drugs to RxNorm, diseases to SNOMED-CT, genes to HUGO gene symbol and so on. Once the PGx associations were normalized and scientific evidences were identified from SemMedDB, we built the CPN. Figure 1 presents the architecture developed for the CPN construction. More details about each step and case studies will be described in the following sections.

### Cancer based PGx association identification

To extract cancer based PGx associations, we first manually collected 160 distinct NCI cancer terms called as seeds from the NCI Cancer List. Then we performed an iterative search to identify PGx associations related to these seeds from PharmGKB. This search was not terminated until fourth-degree concepts that are four nodes away from the seeds have been extracted. More specifically, starting from those seeds, we searched for first-degree concepts that are directly connected to the seeds, then we retrieved second-degree concepts that are neighbors of the first degree concepts, followed by locating third-degree concepts that are neighbors of the second-degree concepts, then the fourth–degree concepts. We iteratively extracted associations related to these seeds from fields listed in Table 1. For instance, beginning with the seed “Urinary Bladder Neoplasms”, we can iteratively find associations, including “rs762551” - “Urinary Bladder Neoplasms”, “Arthritis, Rheumatoid” - “rs762551”, “CYP1A2” - “Arthritis, Rheumatoid”, and “Olanzapine” - “CYP1A2”, which are shown in Table 1. These pairs are as building blocks being used for constructing the CPN. Besides drug, disease and gene, we also extracted haplotype and SNP information that exist in the PharmGKB relationship file. To reflect an assumption that concepts with shorter distance to the seeds might have stronger associations with these seeds, we assigned different weight scores to PGx concepts based on their degrees. The first-degree concept was conferred with a higher weight score of “4”, then the second degree with “3”, the third degree with “2” and the fourth degree with “1”.

Additional PGx information available from the GWAS Catalog and the FDA biomarker table has also been extracted. We manually identified the seeds in the GWAS Catalog based on the NCI cancer terms. We then extracted the PGx associations related to the seeds from fields of “Disease/Trait”, “Reported Gene(s)” and “SNPs” in the GWAS Catalog. It is worthy to note that we did not perform an iterative search to find indirect associations from GWAS Catalog, as we were only interested in the associations extracted from this Catalog co-occurring in the PharmGKB. In parallel, we extracted PGx pairs between “Oncology” drugs and associated genes from the FDA biomarker table.

### Cancer based PGx association normalization

We normalized disease terms by SNOMED-CT [18], drugs by RxNorm [19], genes by the Human Genome Organization (HUGO) [21] gene symbols, SNP by the National Center for Biotechnology Information [22] reference SNP ID number (rsID). Genes, SNPs, haplotypes derived from the three resources have already been represented in standard forms. Therefore, no additional normalization process has been performed accordingly. In this study, we primarily focused on the normalization for drug and disease terms.

#### A. Disease term normalization

PharmGKB provides manual annotations for disease terms with normalized vocabularies, including SNOMED-CT [18], Medical Subject Headings (MeSH) [23], Unified Medical Language System (UMLS) [24], etc., which are available in the downloadable Disease file. However, the mapping to SNOMED-CT is incomplete. There is no SNOMED-CT code available for cancer terms in GWAS catalog. Thus, we normalized disease terms that are without SNOMED-CT codes by employing the NCBO Bioportal REST service [17] programmatically. A Java program has been written to automatically invoke this REST service and parse the XML file as output to retrieve SNOMED-CT codes. Note that we specified “isexactmatch = 1” as one of the input parameters when executing the NCBO REST service. That is to say, the mapped SNOMED-CT terms are exactly matched to the input disease names, thus, no additional evaluation is needed to validate the mapping performance afterwards. We manually checked and mapped the unmapped disease terms to SNOMED-CT with their synonyms.

#### B. Drug term normalization

The same mapping strategy has been applied to drug terms, 1) we reused the normalized terms from the PharmGKB; 2) the NCBO Bioportal REST service was invoked to retrieve RxNorm Concept Unique Identifiers (RxCUIs) for those PharmGKB drugs and the drugs from the FDA biomarker table (no drug information in the GWAS catalog) that are without RxCUIs; 3) manual annotation was performed for unmapped drugs.

Two authors (LW, QZ) had reviewed and evaluated the mappings, and finalized the mapping lists for further CPN construction.

### Scientific evidence identification

To insert scientific evidence, namely, published studies to support PGx associations presented in the CPN, we searched for SemMedDB accordingly. Besides PubMed references existing in the PharmGKB, we searched for PGx associations from the GWAS catalog and the FDA biomarker table against SemMedDB.

### Cancer based PGx network construction

Once the cancer based PGx associations were identified, we linked concepts occurring across three resources to construct the CPN. In the CPN, the nodes correspond to individual cancer based PGx concepts including drug, gene, disease, SNP and haplotype. The edges correspond to PGx associations. Table 2 shows the types of PGx associations contained in the CPN.

**Table 2:** **Types of association available in the CPN**

Pairs Resources	Drug-gene	Drug-haplotype	Drug-disease	Drug-SNP	Drug-drug	Disease-SNP	Disease-hyplotype	Gene-disease	Gene-gene	Gene-SNP
PharmGKB	√	√		√	√	√	√	√	√	
GWAS catalog						√		√		√
FDA biomarkers	√		√							

## Results

### Cancer based PGx association identification

#### A. PharmGKB

Total 38 distinct seeds have been identified from the PharmGKB. Accordingly, we have extracted 2,964 concepts that are associated with these seeds, corresponding to 13,221 PGx pairs. Among these pairs, there are 402 drugs, 205 diseases, 825 genes, 1333 SNPs and 199 haplotypes.

Table 3 shows results of PGx associations extracted from the PharmGKB. For example, there are 38 seeds (cancer terms) associated with 393 Disease-Gene pairs, 37 Disease-Haplotype pairs and 530 Disease-SNP pairs. The numbers shown in Table 3 are unique.

**Table 3:** **Results of PGx association extraction from the PharmGKB**

Degree of concepts	Number of concepts	No. of pairs							
		Disease-gene	Disease-haplotype	Disease-SNP	Drug-gene	Drug-haplotype	Drug-SNPs	Drug-drug	Gene-gene
Seeds	38	393	37	530	0	0	0	0	0
1	605	1018	50	1155	1827	77	1607	0	195
2	735	1700	278	2483	2972	974	3716	1	944
3	2646	1705	277	2492	2965	974	3710	1	982
4	1196	0	0	0	0	0	0	0	0
Total	2964	1723	277	2500	3012	974	3718	1	1016

#### B. FDA biomarkers and GWAS catalog

We manually identified 42 cancer drugs from the FDA biomarker table. As some of drugs are associated with multiple genes, total 55 drug and gene pairs corresponding to 44 genes were extracted.

We extracted 31 cancer terms from the GWAS catalog, of which there are 2455 PGx pairs corresponding to 720 genes and 598 SNPs.

### Cancer based PGx association normalization

Among 402 drugs extracted from the PharmGKB in this study, RxCUIs are available for 323 drugs. For the rest of 79 drugs without RxCUIs, 53 were mapped to RxNorm by invoking the NCBO REST service programmatically. For 205 PharmGKB disease terms being used in this study, SNOMED-CT codes are available for 186 disease terms. Another 10 diseases were mapped to SNOMED-CT by invoking the NCBO REST service programmatically. Of 42 drugs from the FDA biomarker table, 41 were mapped to RxNorm by using NCBO REST service. Of 31 cancer terms identified from the GWAS Catalog, 29 were mapped to SNOMED-CT by the NCBO REST service. Furthermore, we manually mapped 5 drugs and 8 diseases to the standards accordingly.

In summary, 394 out of 416 (94.7%) unique drug concepts have been mapped to RxNorm, and 215 out of 218 (98.6%) unique disease concepts been mapped to SNOMED-CT. Reasons for the failed mapping will be discussed in the discussion section.

### Scientific evidence identification

Besides PubMed references existing in the PharmGKB, 19 PGx pairs corresponding to 16 drugs and 13 genes in the FDA biomarker table were retrieved to be with PubMed IDs and 6 predicates including “COEXISTS_WITH”, “compared_with”, “higher_than”, “INHIBITS”, “INTERACTS_WITH” and “USES” from the SemMedDB. Meanwhile, total 253 PGx pairs (24 diseases and 89 genes) from the GWAS Catalog were retrieved to be with PubMed IDs and 8 predicates including “AFFECTS”, “ASSOCIATED_WITH”, “AUGMENTS”, “CAUSES”, “NEG_ASSOCIATED_WITH”, “NEG_PART_OF”, “PART_OF” and “PREDISPOSES” from the SemMedDB.

### Cancer based PGx network (CPN)

The CPN contains 4,342 distinct nodes and 15,600 pairs in total. We explored Cytoscape [25] to visualize the CPN. A sub-network extracted from the CPN specifically for “urinary bladder cancer” is shown at the left lower corner of Figure 1.

### Case studies

The CPN provides comprehensive PGx information to support advanced cancer relevant research. Specifically, we can identify possible drug repurposing candidates from the CPN by utilizing network analysis approaches. The below two case studies illustrate the capability of the CPN for drug repurposing. It is worthy to note that we manually identified relevant literatures to further evaluate the findings produced in these two case studies and the feasibility of this present study for drug repurposing. However, the ultimate goal of this study is to identify novel drug repurposing candidates that are without supportive scientific evidences, and they will attract interests of chemists and/or biologists for further experimental evaluation.

#### A. Case study 1

Paclitaxel is used to treat Kaposi’s sarcoma, as well as the lung, ovarian, and breast cancer, as documented in the “Indications & Usage” section of the structured product label [26]. In this case study, we were interested in revealing the new indications of Paclitaxel from the CPN. We searched the CPN for Paclitaxel with RxCUI = “56946” and identified relevant disease concepts that are at most 3 nodes away from the Paclitaxel. More specifically, we searched for direct and indirect disease associations that are relevant to Paclitaxel and those disease nodes are at most 3 nodes away from Paclitaxel. In total, there are 70 concepts directly associated with Paclitaxel, 399 concepts including 110 disease concepts that are two nodes away from Paclitaxel, and 1689 concepts including 110 disease concepts that are three nodes away from Paclitaxel. To further evaluate and determine the possible novel indications and the appropriateness of our approach, we manually sought scientific evidences from PubMed literatures to support new indications inferred from the CPN. As a result, 20% newly identified indications including Alzheimer Disease, Asthenia, Leukemia, etc. for Paclitaxel are supported by published studies. To detail our approach, Alzheimer Disease as one novel indication identified for Paclitaxel from the CPN is shown as below.

**Figure 2:**
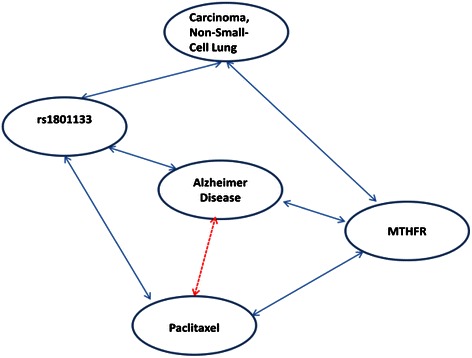
**A sub-network of Paclitaxel taken from the CPN.** Blue solid lines indicate the direct association existed in the CPN, while the red dotted line indicates the indirect inference applied in this case study.

“MTHFR” and **“**rs1801133” are the two direct nodes connected to Paclitaxel, subsequently “Alzheimer Disease” with SNOMED-CT code, “26929004” has been identified via the above two nodes, as shown in Figure 2. rs1801133 is encoding a variant in the MTHFR gene, which encodes an enzyme involved in folate metabolism [27]. Then associations of Paclitaxel-MTHFR-“Alzheimer Disease”, can be further validated by literatures as follows, 1) Paclitaxel enhanced the inhibition of MTHFR by antisense or small molecules, which decreases tumor growth [28]; 2) The severity and biochemical risk factors of Alzheimer’s disease may be influenced by the MTHFR 677 T allele in an Egyptian population [29] and the association between MTHFR A1298C polymorphisms as a possible risk factor and Alzheimer’s disease was verified [30].

By analyzing the CPN, Paclitaxel is related to “Alzheimer Disease” via gene MTHFR and SNP “rs1801133”. In addition, evidences are mounting in the literature that Alzheimer disease may be a new indication of the cancer drug Paclitaxel, for example Paclitaxel may rescue neurons from undergoing hallmark tau-induced Alzheimer disease cell pathologies [31] and Paclitaxel has the potential to treat Alzheimer disease [32]. That is to say,Paclitaxel may be a potential drug repurposing candidate for the treatment of Alzheimer Disease.

#### B. Case study 2

Capecitabine is originally indicated for the treatment of breast cancer and colorectal cancer as stated in the drug label [33]. In this case study, we aimed to seek alternative indications for Capecitabine. We searched for Capecitabine with RxCUI “194000” from the CPN to identify novel indications. In total, there are 120 disease nodes that are at most 3 nodes away from Capecitabine. Of these 120 diseases, 12 possible novel indications including Hyperbilirubinemia, Mesothelioma, Bladder Neoplasm, etc. associated with Capecitabine are supported by published studies. The following example illustrates the identification process of the new indication, bladder neoplasm for Capecitabine.

**Figure 3:**
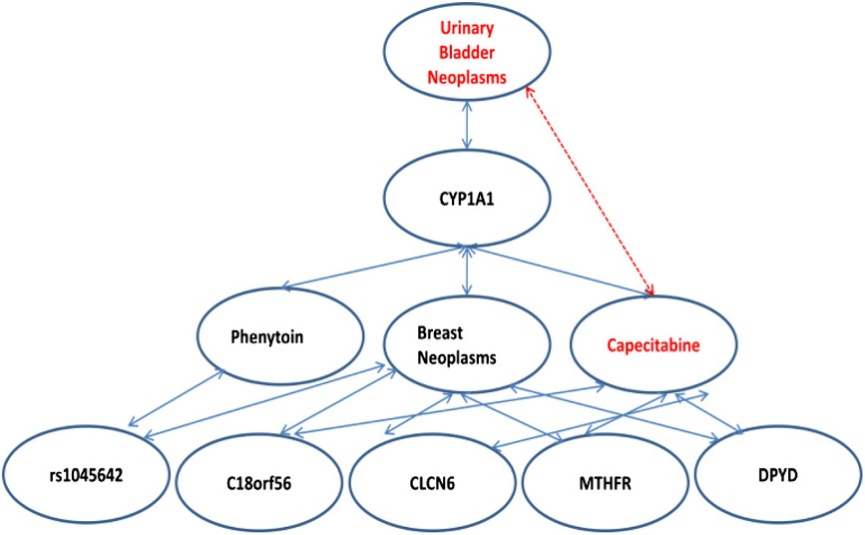
**A sub-network of Capecitabine taken from the CPN.** Blue solid lines indicate the direct association existed in the CPN, while the red dotted line indicates the indirect inference applied in this case study.

From the CPN 50 directly relevant nodes have been retrieved for Capecitabine including the gene CYP1A1, from which “Urinary Bladder Neoplasms” have been identified subsequently. A sub-network of Capecitabine visualized by Cytoscape in the CPN is shown at the right lower corner in Figure 1, where the edges in red indicate all associations with Capecitabine, and the green edges indicate DPYD and C18orf56 are linking to Capecitabine respectively. The zoomed out network is shown in Figure 3. The association between “Urinary Bladder Neoplasms” and “Capecitabine” could be inferred through multiple paths as shown in Figure 3. Among all paths between these two, the shortest path is Capecitabine-CYP1A1-Urinary Bladder Neoplasms, of which the association could be proved by literatures: (1) “CYP1A1 rs1048943 A > G (Ile462Val) polymorphism is a potential prognostic marker for survival outcome after docetaxel plus capecitabine chemotherapy” [34]; (2) active CYP1A1 and CYP1B1 overexpression is revealed in bladder cancer [35]; (3) the combination of Capecitabine and radiation therapy offers a promising treatment option for bladder cancer patients who are not candidates for surgery or cisplatin-based chemotherapy [36]; (4) a patient with metastatic bladder cancer responded well to second-line capecitabine with a clinically meaningful progression-free survival [37]. Through this validation chain, the inference that the breast and colorectal cancer drug, “Capecitabine” might be used for urinary bladder cancer could be made. Evidently urinary bladder cancer may be a novel indication of Capecitabine via the network-based analysis of the CPN.

## Discussion

### Benefits gained from the CPN

#### A. Supporting further data integration

Data integration is essential in the big data era. It is important to aggregate different pieces of data from different areas to solve fundamental scientific questions. Particularly, in this study we have integrated data from various PGx data resources and built a cancer based PGx data repository. The concepts (nodes) included in the CPN were normalized with multiple standard biomedical terminologies and domain standards. Once the normalization task is accomplished, more relevant data can be deposited and integrated into the CPN, such as Electronic Medical Records (EHRs), DrugBank [38] and KEGG [39]. Besides a majority portion (99.4%) of the concepts has been normalized, about 0.6% of concepts was failed to be normalized. The reason of failure is in two folds. First, chemical IUPAC names were used as drug names in the PharmGKB, which were not included in RxNorm, e.g., “1-methyloxy-4-sulfone-benzene”. Second, drug class names were being used, such as “Analgesics and Anesthetics” and “Antiinflammatory and Antirheumatic Products”. In terms of diseases, the names were either presented too broadly, such as, “Substance-Related Disorders” or too narrowly, such as “Therapy Related Acute Myeloid Leukemia”, so that they cannot be mapped to SNOMED-CT.

#### B. Supporting oncology based drug discovery

PGx data including the detailed information for drugs, diseases, genes, SNPs, etc., has been regarded as a basis for individualized medicine. While generic PGx data could be obtained publicly, drug, disease, gene, SNP and haplotype resources have not, as yet, been well-integrated to support the oncology based drug discovery. With various association types including Disease-Gene, Drug-Gene, etc. as shown in Table 2, the CPN can serve as a highly relevant cancer knowledge base and a valuable platform for oncology based research on drug repurposing. Thus, it would result in the shortening of the entire process for drug development, as our case studies have successfully proved such capability of the CPN. Additionally two advantages inherent in the CPN will strengthen its application in drug repurposing, including: 1) the CPN contains both direct and indirect cancer based PGx associations, thus, more drug candidates can be identified via automated inference; 2) a majority of concepts contained in the CPN are normalized with standard vocabularies, which enables further integration with other relevant resources to support more novel indication identifications.

### Limitation and future study

#### A. Path ranking

The current version of the CPN includes cancer based PGx information extracted from three major PGx resources. Although only 38 cancer terms have been found in the PharmGKB, 42 cancer drugs identified from the FDA biomarker table, and 31 cancer terms found from the GWAS catalog, the total number of nodes and edges of the CPN is 19,942, as we included all associations up to four nodes away from the cancer seeds. In this study, we focused on the CPN construction and the demonstration of the capability of the CPN. Path ranking to output a ranked list of paths that are associated with specific concepts from the CPN was out of scope of this study. However, when we conducted case studies, in order to filter out the most significant paths based on the queries, some initial ranking rules have been applied. For example, weight scores according to the degrees of concepts, path length, and VIP pairs from the PharmGKB have been applied for path ranking. In the future study, we will incorporate these rules with other ranking methods, such as PageRank [40], and genetic association p-values derived from GWAS [11], to output the most correlated paths for a particular query.

#### B. Disambiguating drug-disease association

Detailed information on specifying drug and disease association is critical for drug repurposing, as we have to determine whether this drug is used to treat this disease or this drug may cause such a disease as an adverse drug event. Consequently, the novel indication may be identified for this drug for further evaluation. In this study, all drug and disease associations were directly extracted from the original resources, no additional step has been applied to disambiguate such associations. In our previous study, we have employed NDF-RT and SPLs to annotate drug and disease relationships in the PharmGKB [41]. We will apply the annotation results [41] along with the existing annotations from NDF-RT, ADEpedia [42], LinkedSPLs [43] into the future study, inserting a particular tag for differentiating indications and adverse drug events.

#### C. Scientific evidence identification

The established CPN is supported by published studies, PubMed references extracted from SemMedDB. However, not all CPN associations have been assigned with pubmed IDs. 36 PGx pairs from the FDA biomarker table and 2202 PGx pairs from the GWAS Catalog were not mapped. SemRep, a rule-based semantic interpreter extracting predicates in Pubmed references being applied by SemMedDB, has shown its precision for gene-disease relations as 76% [44]; the precision and recall for pharmacogenomics as 73% and 55% [45] respectively. Thus we doubt that all association presented in this study have been extracted and included in SemMedDB. On the other hand, we performed direct mapping with FDA biomarker table and the GWAS Catalog that may cause missing mappings. In the future, we would use machine learning and natural language processing (NLP) to identify more associations on the basis of existing PubMed references.

#### D. Data integration

In this preliminary study, we extracted and integrated three well-known PGx resources to build the CPN. To make the CPN more informative, we will extract further cancer based PGx information from other public PGx resources, such as DrugBank, KEGG, etc., them and integrate into the CPN. Meanwhile, we will identify PGx associations from pathways, and apply NLP [46] tools and algorithms to automatically extract such associations from literatures periodically. The ultimate goal will be leveraging semantic web technologies (SWT) [47] to present such comprehensive cancer based PGx information in RDF [48] or OWL [49], which can support automated inference for drug repurposing.

## Conclusions

In this study we have integrated three existing PGx resources into the CPN, which is supported by published studies, PubMed references extracted from SemMedDB. The established CPN offers comprehensive cancer based PGx information to support cancer orientated research, especially for drug repurposing, the potential of which has been successfully demonstrated by case studies.

## Abbreviations

PGx: Pharmacogenomics
SNPs: Single nucleotide polymorphisms
CPN: Cancer based PGx network
PharmGKB: Pharmacogenomics knowledge base
GWAS: Genome-wide association studies
OWL: Web ontology language
NCI: National Cancer Institute
SNOMED-CT: Systematized nomenclature of medicine-clinical terms
NCBO: National Center for Biomedical Ontology
MeSH: Medical subject headings
UMLS: Unified medical language system
rsID: Reference SNP ID number
